# The Biological Clock–Mitochondria Axis in the Liver: From Molecular Mechanisms to Metabolic Disease

**DOI:** 10.3390/biology15141197

**Published:** 2026-07-20

**Authors:** Virginia Manuti, Emanuele Murgo, Anna Alessia Saponaro, Umberto Sfregola, Moris Sangineto, Rosanna Villani, Gaetano Serviddio, Gianluigi Mazzoccoli, Tommaso Colangelo

**Affiliations:** 1 Cancer Cell Signaling Unit, Fondazione IRCCS “Casa Sollievo della Sofferenza”, Viale Padre Pio 7, 71013 San Giovanni Rotondo, Italy; virginiamanuti@gmail.com (V.M.); a.saponaro@operapadrepio.it (A.A.S.); 2 Center for Research and Innovation in Medicine (C.R.E.A.T.E.), Department of Medical and Surgical Sciences, University of Foggia, Viale Luigi Pinto, 71122 Foggia, Italy; umberto_sfregola.627040@unifg.it (U.S.); moris.sangineto@unifg.it (M.S.); rosanna.villani@unifg.it (R.V.); gaetano.serviddio@unifg.it (G.S.); 3 Chronobiology Laboratory, Fondazione IRCCS “Casa Sollievo della Sofferenza”, Viale Padre Pio 7, 71013 San Giovanni Rotondo, Italy; g.mazzoccoli@operapadrepio.it; 4 C.U.R.E. (University Center for Liver Disease Research and Treatment), Department of Medical and Surgical Sciences, University of Foggia, Viale Luigi Pinto, 71122 Foggia, Italy

**Keywords:** circadian clock, hepatic mitochondria, mitochondrial dynamics, NAD^+^–sirtuin axis, MASLD, MASH, chronotherapy, chronopharmacology

## Abstract

The liver ranks among the peripheral organs exhibiting the most robust circadian rhythmicity, with glucose homeostasis, lipid metabolism, and bile acid turnover governed by tightly phased diurnal oscillations. Mitochondria execute these programs, their output coordinated with the hepatocyte circadian state. The mitochondrial network undergoes dynamic remodeling across the 24 h cycle, encompassing oscillatory changes in bioenergetics, fusion–fission balance, and quality control. This interplay is bidirectional: core clock components drive rhythmic remodeling via cyclin-dependent kinase 1/mitogen-activated protein kinase (CDK1/MAPK)-dependent phosphorylation of dynamin-related protein 1 (DRP1) and the NAD^+^–SIRT1/SIRT3 axis, while retrograde signals modulate clock amplitude and entrainment. Circadian disruption is associated with mitochondrial dysfunction implicated in MASLD onset and progression to MASH and HCC, though this evidence remains largely correlative and derives predominantly from rodent models. This review integrates clock–mitochondria coupling with metabolic liver disease. Restoring this coupling has been proposed as a candidate chronotherapeutic strategy, supported by preliminary rhythmicity data in primary human hepatocytes and a hepatocellular carcinoma cell line, though causal validation in healthy human liver is lacking. Time-restricted feeding, NAD^+^ precursors, PPAR agonists, and ACC inhibitors converge on clock-regulated pathways and may benefit from circadian-informed timing, though this remains unverified.

## Simple Summary

The human body operates on an endogenous 24 h timekeeping system, and the liver is among the peripheral organs most tightly entrained to this circadian program. Hepatic mitochondria, the primary sites of oxidative metabolism, undergo rhythmic remodeling in morphology and bioenergetic output under transcriptional control of the core molecular clock. Perturbation of this temporal coordination, through circadian misalignment, shift work, or loss-of-function clock mutations, renders the liver susceptible to steatosis, necro-inflammation, fibrosis, and hepatocellular carcinoma. The mechanistic evidence linking circadian and mitochondrial dysregulation to MASLD/MASH pathogenesis remains predominantly derived from rodent models and is largely correlative in nature. Accordingly, this review consolidates the available evidence into a disease-stage-specific conceptual framework, intended to inform, rather than substantiate, hypotheses for future validation in human tissue. This review delineates the molecular mechanisms underlying clock–mitochondria coupling in the healthy liver, the pathophysiological consequences of their uncoupling across metabolic liver disease, and the chronotherapeutic potential of nutritional and pharmacological interventions. Circadian alignment of treatment schedules may represent a cost-effective strategy to enhance therapeutic efficacy without novel drug development.

## 1. Introduction

The circadian clock constitutes an evolutionarily conserved molecular timing system that aligns physiological processes with the 24 h light–dark cycle. In mammals, this system is organized hierarchically: a central pacemaker residing in the suprachiasmatic nucleus (SCN) coordinates peripheral oscillators via neuroendocrine and metabolic cues [1,2], whereas the hepatic oscillator is predominantly entrained by feeding–fasting cycles rather than photic signals [3,4]. Multi-omics investigations have established that circadian regulation operates across multiple layers of gene expression, encompassing the transcriptome and metabolome, and governs hepatic glucose homeostasis, lipogenesis, bile acid biosynthesis, and xenobiotic detoxification [5,6].

Mitochondria are indispensable effectors of hepatic metabolic function, and a growing body of evidence demonstrates that they are subject to pervasive circadian regulation. The dynamin-related GTPase DRP1 undergoes rhythmic phosphorylation at Ser-637, driving cyclic transitions between mitochondrial fission and fusion states that are temporally coupled to diurnal oscillations in oxidative phosphorylation (OXPHOS) capacity [7]. Hepatocyte-specific deletion of Bmal1 abolishes circadian cycling of DRP1 phosphorylation, mitochondrial network dynamics, and respiratory chain activity [8]; furthermore, Drp1 expression is itself subject to rhythmic post-transcriptional regulation by CLOCK [9]. Critically, this relationship is bidirectional: pharmacological inhibition of DRP1 lengthens circadian period and attenuates amplitude of core clock oscillations [7,10], establishing a closed regulatory loop rather than a unidirectional hierarchy.

The pathophysiological relevance of this axis is most apparent in the context of metabolic liver disease. MASLD, redefined by the 2023 multi-society consensus [11], affects up to 38% of the adult population [12,13] and progresses through MASH, fibrosis, and HCC. Mitochondrial dysfunction represents a hallmark feature across all disease stages [14,15], and circadian disruption has been shown to exacerbate hepatic pathology in rodent models and to correlate with disease severity in human cohorts [16,17,18]. Rhythmic oscillation of NAD^+^ bioavailability and the consequent circadian deacetylation of mitochondrial protein complexes by SIRT1/SIRT3 constitute an additional mechanistic layer within this regulatory axis [19,20].

Despite the mechanistic evidence linking circadian dysregulation to hepatic disease, chronotherapeutic strategies remain profoundly underexplored in hepatology: fewer than 0.16% of registered clinical trials incorporate time-of-day as an experimental variable, and no trial to date has targeted a liver-specific indication [21]. Nevertheless, several agents with established or emerging efficacy in MASH, including time-restricted feeding, NAD^+^ precursors, PPAR agonists, ACC inhibitors, MSDC-0602K, resmetirom, and FGF21-based therapies, exert their effects through rhythmically regulated molecular pathways [22], raising the prospect of chronopharmacological optimization.

While several reviews have addressed circadian–mitochondria interplay in general terms or hepatic circadian physiology independently of mitochondrial function, this review specifically integrates clock–mitochondria coupling within the metabolic liver disease, while explicitly delineating the associative versus causal strength of the current evidence base.

In this review, we systematically examine the circadian mechanisms governing hepatic mitochondrial function and delineate how their disruption contributes to metabolic liver disease progression.

## 2. The Biological Clock in the Liver

The mammalian timing system is hierarchical. The central pacemaker, the SCN, receiving light input through the retinohypothalamic tract, synchronizes peripheral oscillators, such as the liver, using neuronal, endocrine, and metabolic outputs [1,2]. However, the machinery of the circadian clock is the same for all cells—TTFLs producing sustained rhythms of a ~24 h period [1,23]. In canonical TTFL, CLOCK (or its paralog NPAS2) forms a heterodimer with BMAL1 and binds E-box motifs in Per1, Per2, Cry1, and Cry2 gene promoters. PER and CRY accumulate in the cytoplasm and return to the nucleus once their concentration rises high enough. In the nucleus, PER and CRY inhibit the activity of the CLOCK: BMAL1 complex, shutting off their own transcription. Finally, degradation of PER and CRY by F-box protein-dependent proteasome leads to activation of the TTFL and restarts the cycle [23]. Another stabilizing loop is based on competition between two families of nuclear receptors: REV-ERBα/β (NR1D1, NR1D2) and RORα/β/γ. These factors compete for the binding sites within the Bmal1 promoter and suppress or activate it, respectively [23]. In addition to functioning as clock components, some of the core TTFL constituents participate in cell-cycle regulation and autophagy [23].

The hepatic circadian clock exhibits a distinctive characteristic in that feeding cues, rather than the light–dark cycle, serve as its primary zeitgeber. Experimental studies in nocturnal rodents have demonstrated that restricting food availability to the inactive (light) phase induces an approximately 12 h phase shift in the liver clock relative to the SCN, accompanied by a corresponding shift in the peak expression of most clock genes and clock-controlled transcripts [3,4]. Remarkably, even in Cry1^−/−^; Cry2^−/−^ mice, which lack a functional molecular circadian oscillator, the imposition of a feeding rhythm is sufficient to generate rhythmic patterns of hepatic gene expression, highlighting the dominant role of nutrient timing in regulating liver transcriptional programs [4].

However, compared to the liver, the impact of similar feeding manipulations on circadian rhythmicity in other peripheral organs, such as the kidneys, heart, and lungs, is relatively minimal, indicating that the liver’s circadian pacemaker is highly responsive to metabolic factors [3]. Furthermore, studies employing hepatocyte-specific restoration of clock function have revealed that hepatocytes possess the capacity to synchronize their oscillatory activity independently of other peripheral clocks, with feeding schedules further strengthening this intrahepatic synchronization [24]. Collectively, these findings indicate that individual tissues utilize distinct entrainment pathways that connect external stimuli to the core molecular clock, reflecting their specialized physiological and metabolic functions [3,5].

Twenty years of omics studies have resulted in a fairly comprehensive view of how ubiquitous the hepatic clock is. Rhythms have been described at the transcriptomic, cistromic, proteomic, phosphoproteomic, acetylproteomic, metabolomic, and lipidomic levels [5,6,25,26]. A few metabolic sensors link the cellular energy status to the clock via positive and negative feedback. The NAD^+^-dependent deacetylase SIRT1 displays circadian rhythms as a response to NAD^+^ oscillations due to oscillations in the NAD^+^ production, NAD^+^ being produced via NAMPT, which regulates NAD^+^ biosynthesis in a circadian fashion; SIRT1 then deacetylates PER2 and BMAL1 to modulate the period and amplitude of the hepatic clock [27]. AMPK, activated by a low ATP/AMP ratio, phosphorylates and degrades CRY1, thus affecting the speed of the clock [28]. The coactivator of mitochondrial gene expression, PGC-1α, also acts on the clock gene [29]. They are not merely the downstream effectors of the clock: they are embedded in regulatory feedback loops ensuring the robustness and metabolic responsiveness of the clock [5,6].

Downstream, the hepatic clock regulates glucose metabolism, lipogenesis, bile acid synthesis, and xenobiotic metabolism. In hepatocytes, Bmal1 deletion results in fasting hypoglycemia and loss of glucose transporter 2 rhythmicity, demonstrating how essential the role of the liver clock is in glucose regulation under post-absorptive conditions [30]. Recent proteomic studies have also demonstrated that disruptions of the circadian system via external factors lead to widespread remodeling of the proteome in the liver, and thus lipid and glucose metabolism are also altered in ways that are more complex than merely the change at the transcript level [31]. As a result of circadian disruption caused by shift work, chronic jet lag, high-fat diet, and knockout of key circadian proteins, the development of a group of diseases occurs, including obesity, type 2 diabetes, MASLD/MASH, and HCC [5,6,32].

## 3. Circadian Control of Mitochondrial Dynamics

The mitochondrion is an active organelle. Its reticulum is continuously remodeled by the actions of fusion and fission, and the dynamics of these processes define the structure and functionality of this organelle. The processes of mitochondrial fusion are mediated by the mitofusin MFN1/2 proteins located in the outer mitochondrial membrane and by the OPA1 protein in the inner mitochondrial membrane. These processes result in the formation of long, tubular mitochondria with high oxidative phosphorylation and ATP production capacity. Mitochondrial fission is mediated by DRP1GTPase, which targets the constrictions [33,34]. Strong evidence for the circadian regulation of mitochondrial morphology was provided by Schmitt et al., who employed confocal microscopy to demonstrate a robust 24 h oscillation in mitochondrial network architecture in synchronized human skin fibroblasts [7]. Sixteen hours following synchronization, mitochondria exhibited a highly interconnected and tubular morphology, coinciding with maximal ATP production and respiratory activity. By contrast, 28 h after synchronization, the network became fragmented and bioenergetic output reached its lowest level [7]. Similar rhythmic changes have been observed in vivo. At circadian time (CT) 0, corresponding to the onset of the subjective rest phase, mitochondria display a predominantly tubular morphology in both mouse hippocampal CA1 neurons and hepatocytes. In contrast, at CT12, marking the beginning of the active phase, the mitochondrial network becomes fragmented. Notably, in mPer1/mPer2 double-knockout mice, mitochondria remain constitutively fragmented throughout the circadian cycle, indicating a critical role for PERIOD proteins in maintaining rhythmic mitochondrial remodeling [7].

Significantly, these cycles do not correlate with cell cycle phases either. Similar patterns of cycling have been detected in post-mitotic cells [7] and also in AraC-treated fibroblasts, proving that circadian modulation of mitochondrial morphology operates independently of cell division [7].

Several independent studies have subsequently reinforced these observations, collectively demonstrating a critical role for the circadian clock in regulating mitochondrial dynamics through multiple molecular mechanisms. Jacobi et al. reported that hepatocyte-specific deletion of Bmal1 abolishes rhythmic mitochondrial remodeling and impairs respiratory chain function in vivo [8]. This phenotype was accompanied by reduced expression of key genes involved in mitochondrial fission and quality control, including Drp1, Fis1, Pink1, and Bnip3. Building upon these findings, Xu et al. [9] identified a post-transcriptional mechanism through which CLOCK regulates mitochondrial dynamics. In the mouse liver, CLOCK interacts with the RNA-binding protein PUF60 to promote Drp1 mRNA degradation. Consequently, ClockΔ19 mutant mice exhibit excessive mitochondrial fragmentation, elevated reactive oxygen species (ROS) production, and impaired aerobic metabolism. This resembles a non-alcoholic fatty liver disease (NAFLD) phenotype, and can be ameliorated by treatment with the DRP1 inhibitor Mdivi-1 [9].

Further evidence for the importance of DRP1-mediated mitochondrial remodeling was provided by Steffen et al., who demonstrated that hepatocyte-specific deletion of Drp1 exacerbates NASH progression and activates the mitochondrial integrated stress response, indicating that appropriate mitochondrial fission is required to maintain hepatic homeostasis and limit disease progression [35]. In addition, recent reviews have consolidated these findings and highlighted that proteins involved in mitochondrial fission and mitophagy exhibit pronounced diurnal oscillations in wild-type mouse liver, whereas these rhythms are markedly attenuated in Bmal1-deficient animals [36,37]. Moreover, reduced Bmal1 expression directly compromises BNIP3-mediated mitophagy, thereby contributing to mitochondrial dysfunction and cellular stress.

Collectively, these studies indicate that the circadian clock regulates both the structural organization and turnover of the mitochondrial network through multiple complementary mechanisms, including transcriptional control of genes involved in mitochondrial fission and mitophagy, post-transcriptional regulation of Drp1 mRNA stability, and post-translational modification of DRP1 activity.

Among these regulatory mechanisms, the post-translational control of DRP1 has been particularly well characterized. Although total DRP1 protein abundance remains relatively constant throughout the day, phosphorylation of DRP1 at Ser637 exhibits a robust circadian rhythm in synchronized fibroblasts and mouse brain tissue [38,39]. Phosphorylation at this residue suppresses DRP1 GTPase activity by disrupting the interaction between its GTPase and GED domains, thereby favoring mitochondrial fusion and the maintenance of an interconnected mitochondrial network [38,39]. Notably, this rhythmic phosphorylation pattern is completely abolished in mPer1/mPer2 double-knockout mice [7].

The phosphorylation status of Ser637 is determined by the balance between protein kinase A (PKA) and calcineurin. PKA phosphorylates and inhibits DRP1, promoting mitochondrial fusion, whereas calcineurin, which is itself subject to circadian regulation, dephosphorylates DRP1 and stimulates mitochondrial fission [40,41]. Consistent with this model, pharmacological inhibition of calcineurin using FK506 eliminates both the circadian rhythm of DRP1 Ser637 phosphorylation and the associated oscillations in mitochondrial network morphology [7]. These findings highlight the central role of post-translational DRP1 regulation in mediating circadian control of mitochondrial dynamics.

## 4. Circadian Control of Mitochondrial Bioenergetics

Beyond its role in regulating mitochondrial morphology, circadian fission–fusion cycling plays a central role in generating rhythmic hepatic bioenergetics. Metabolomic analyses of synchronized human U2OS cells have demonstrated that approximately 29% of detected metabolites exhibit circadian oscillations, including tricarboxylic acid (TCA) cycle intermediates, the GSH/GSSG redox couple, and key energy metabolites such as ATP and NAD^+^/NADH [34]. These findings are consistent with earlier studies in both rodents and humans, highlighting the widespread temporal organization of cellular metabolism [8,9].

Notably, rhythmic ATP production is dependent on an intact circadian clock. Mouse embryonic fibroblasts lacking Per1 and Per2 display persistently low and arrhythmic ATP levels, indicating that ATP oscillations are under clock control [34]. Furthermore, these oscillations originate primarily from OXPHOS rather than glycolysis, as they are abolished by the ATP synthase inhibitor oligomycin but remain unaffected by the glycolytic inhibitor 2-deoxyglucose [34]. Importantly, pharmacological inhibition of DRP1 using P110 or Mdivi-1, as well as genetic deletion of Drp1, eliminates circadian ATP oscillations both in vitro and in vivo, demonstrating that DRP1-dependent mitochondrial fission–fusion dynamics are essential for maintaining rhythmic bioenergetic output [34]. These findings indicate that mitochondrial dynamics are not merely a downstream consequence of circadian regulation but rather a fundamental component of the circadian metabolic program.

The circadian clock regulates mitochondrial dynamics through several well-established metabolic sensors. Pharmacological inhibition of AMPK, SIRT1, or SIRT3 individually abolishes the circadian rhythm of DRP1 Ser637 phosphorylation, positioning these molecules as key intermediates linking the molecular clock to the mitochondrial fission machinery [34]. These sensors are themselves integrated into clock regulatory networks through mechanisms involving CRY phosphorylation and NAD^+^-dependent deacetylation of both clock proteins and mitochondrial targets [6,35,36].

**Figure 1:**
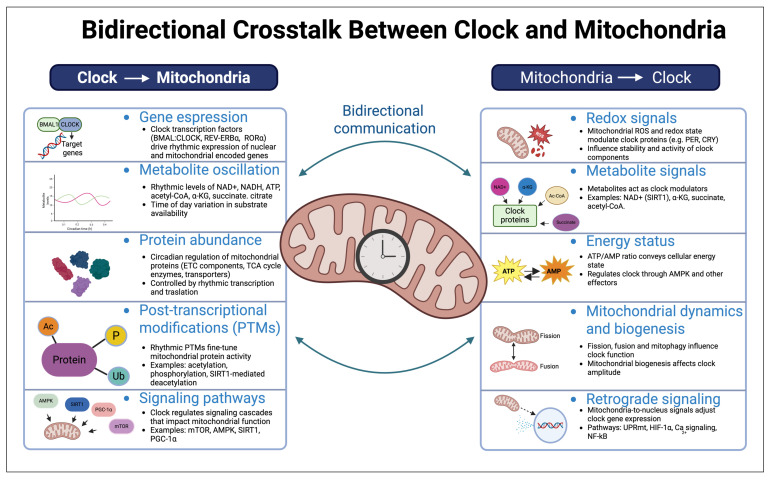
Schematic representation of bidirectional crosstalk between the circadian clock circuitry and mitochondria. Created by Biorender.com.

Importantly, the relationship between the circadian clock and mitochondrial dynamics is bidirectional as illustrated in Figure 1. 

Disruption of mitochondrial remodeling through DRP1 inhibition or calcineurin blockade lengthens the circadian period in Bmal1::luciferase reporter fibroblasts, while Drp1-deficient mouse embryonic fibroblasts exhibit dampened oscillations of Bmal1, Per1, and Per2 expression [34]. These observations suggest that while the circadian clock governs mitochondrial morphology through DRP1-dependent mechanisms, mitochondrial dynamics reciprocally reinforce clock function through ATP- and NAD^+^-dependent signaling pathways involving AMPK and SIRT1.

In the liver, BMAL1 has been shown to coordinate rhythmic mitochondrial remodeling and support metabolic fitness, further emphasizing the importance of the clock–mitochondria axis in hepatic physiology [37].

Beyond these cell-autonomous, DRP1-dependent mechanisms, systemic hormones constitute a complementary, circulating layer through which circadian timing is translated into rhythmic mitochondrial bioenergetic and redox output. Because circulating levels of these hormones themselves oscillate with distinct circadian phases, they convey central SCN timing to hepatic mitochondria in a manner temporally offset from, yet functionally complementary to, the direct AMPK/SIRT-dependent mechanisms described above.

Among these hormonal regulators, cortisol is of primary importance. In the liver specifically, hepatocyte-targeted genetic deletion of the glucocorticoid receptor (GR) disrupts diurnal chromatin binding and rhythmic hepatic gene expression [42,43], and acute hepatocyte-specific GR deletion independently impairs postprandial lipid metabolism and produces a measurable reduction in mitochondrial respiration, most pronounced in the postprandial phase [44]. GR activity is itself gated by the core clock: REV-ERBα physically interacts with GR and licenses its chromatin recruitment in a time-of-day-dependent manner. Glucocorticoid treatment alters mitochondrial mass, the NAD^+^/NADH ratio, and ATP synthesis efficiency differentially between the light and dark phases [45]. This finding directly parallels the clock-dependent ATP and NAD^+^/NADH oscillations described above, but is driven by a systemic rather than cell-intrinsic signal. Chronic excess glucocorticoid signaling has more broadly been linked to mitochondrial stress and metabolic inflexibility across tissues [46]. Cortisol, therefore, shapes hepatic mitochondrial respiratory capacity and biogenesis in a manner that is both liver-specific and strictly time-of-day-dependent, with dysregulated or chronically elevated signaling shifting this same axis toward mitochondrial impairment and increased ROS generation.

Melatonin, secreted in antiphase to cortisol, provides a mechanistically distinct, complementary mode of regulation. Rather than acting transcriptionally, it accumulates directly within the mitochondrial matrix at concentrations reported to exceed circulating plasma levels, where it scavenges reactive oxygen and nitrogen species, stimulates superoxide dismutase, glutathione peroxidase, and catalase activity, and enhances the efficiency of respiratory complexes I and IV [47]. Through this direct biochemical action, melatonin limits nocturnal mitochondrial ROS accumulation, temporally complementing cortisol’s predominantly transcriptional, active-phase mode of action.

Evidence for DHEA and progesterone in this specific context remains comparatively limited. DHEA and its sulfate ester DHEAS do not bind directly to glucocorticoid receptors but instead act as functional antagonists of glucocorticoid signaling, exhibiting concurrent antioxidant properties that may protect against corticosterone-induced cellular stress [48]. This dual action suggests a plausible indirect protective role against cortisol-driven mitochondrial oxidative stress, although a specific mitochondrial mechanism in hepatocytes has not yet been established. Progesterone’s relationship to mitochondrial function is comparatively less defined. Although the translocator protein (TSPO) was historically proposed as the mediator of progesterone-linked mitochondrial cholesterol import for steroidogenesis, TSPO knockout studies have since demonstrated that it is dispensable for this process, with STAR/STARD1 now recognized as the principal cholesterol shuttle mediating the rate-limiting step of steroid hormone biosynthesis. Notably, STAR/STARD1 itself undergoes turnover via a mitophagy-dependent degradation mechanism, situating steroidogenic cholesterol transport within the broader mitophagic regulatory network discussed throughout this review [49]. A direct link between progesterone and hepatic mitochondrial redox regulation, however, remains to be established, and its contribution to this axis is therefore considered minor and mechanistically unresolved relative to cortisol and melatonin.

Collectively, cortisol and melatonin emerge as the two dominant, temporally opposed hormonal regulators of mitochondrial redox state across the 24 h hepatic cycle, acting in parallel with, rather than in place of, the cell-autonomous AMPK/SIRT/DRP1 axis described above. Together, these systemic and cell-intrinsic layers allow hepatocytes to synchronize bioenergetic output not only with the local circadian clock but also with whole-body neuroendocrine timing.

## 5. Disruption of the Clock–Mitochondria Axis in Metabolic Liver Disease

Given the close functional interplay between the hepatic circadian clock and the mitochondrial network, it is unsurprising that disruption of either system adversely affects liver metabolism. Over the past decade, evidence from both animal models and human studies has established a link between circadian dysregulation and the development of metabolic disorders [5,16,32,50]; however, the strength and directness of this evidence differ markedly by species and level of biological organization. Unless otherwise specified, the mechanistic data on mitochondrial dynamics and bioenergetics summarized in this section derive from murine hepatocytes or rodent liver tissue; direct causal evidence in human liver remains limited, and human data discussed below are, unless stated, associative rather than mechanistic (see also Section 7). In mice, both genetic and environmental disruption of the circadian oscillator have been shown to promote insulin resistance, hepatic steatosis, and hepatocarcinogenesis [51,52,53]. Notably, ClockΔ19 mutant mice spontaneously develop steatosis and hyperinsulinemia and exhibit impaired glucose tolerance even when maintained on a standard diet [52]. Similarly, hepatocyte-specific deletion of Bmal1 disrupts rhythmic gluconeogenesis and exacerbates diet-induced liver injury in several models, highlighting the critical role of the hepatic clock in maintaining metabolic homeostasis [54]; as discussed further below, however, this phenotype is not uniform across all Bmal1 loss-of-function models.

In humans, genetic variants of CLOCK have been associated with increased susceptibility to NAFLD, whereas polymorphisms in PER and CRY genes have been linked to obesity and type 2 diabetes mellitus [55,56,57]. Furthermore, large-scale observational studies have identified significant associations between hepatic steatosis and behaviors that promote circadian misalignment, including shift work, chronic sleep restriction, and recurrent jet lag [16,32,58]. Importantly, these associations remain significant after adjustment for potential confounding factors such as dietary habits, body mass index, and physical activity, supporting the notion that circadian disruption itself contributes directly to metabolic disease pathogenesis rather than simply reflecting unhealthy lifestyle behaviors [16,32].

In June 2023, an international multisociety Delphi consensus introduced a revised nomenclature for fatty liver disease, replacing NAFLD with metabolic dysfunction-associated steatotic liver disease (MASLD) and non-alcoholic steatohepatitis (NASH) with metabolic dysfunction-associated steatohepatitis (MASH) [11]. This reclassification was intended to emphasize the underlying metabolic pathophysiology of the disease rather than defining it by the absence of significant alcohol consumption, while also addressing concerns regarding disease-related stigma. Throughout this review, the terms MASLD/MASH and NAFLD/NASH are used interchangeably when discussing earlier studies, as the underlying biological mechanisms remain unchanged.

MASLD is currently the most prevalent chronic liver disease worldwide, affecting approximately 38% of the adult population, with substantially higher prevalence rates among individuals with obesity and type 2 diabetes mellitus [12,13]. Mitochondrial dysfunction represents a hallmark feature throughout the entire disease spectrum. During the early stage of simple steatosis, chronic lipid excess exceeds the β-oxidation capacity of the mitochondrial network, leading to the accumulation of triglycerides, diacylglycerols, and ceramides. In response, mitochondrial mass increases as a compensatory mechanism to maintain respiratory function; however, this adaptation eventually becomes maladaptive, resulting in enhanced electron leakage and excessive production of ROS [14,15].

Notably, similar metabolic alterations are observed under conditions of circadian disruption. Impairment of the core molecular clock leads to persistent mitochondrial fragmentation and loss of rhythmic ATP production [7]. Likewise, hepatocyte-specific deletion of Bmal1 abolishes rhythmic mitochondrial remodeling, compromises respiratory chain efficiency, and impairs overall metabolic fitness [8,54]. Under physiological conditions, the daily alternation between fused and fragmented mitochondrial states enables hepatocytes to adapt their metabolic activity to feeding–fasting cycles. Consequently, disruption of this rhythmic remodeling promotes chronic bioenergetic imbalance. This is characterized by impaired fatty acid oxidation and increased oxidative stress, both central features of MASLD pathogenesis [7,14,15,33,34]. Consistent with these observations, expression of core clock genes, including BMAL1, PER2, and REV-ERBα, is reduced or phase-shifted in liver biopsies from patients with MASLD, and the degree of circadian disruption correlates with histopathological disease severity [16,17,18].

Progression from MASLD to MASH is accompanied by a further deterioration of mitochondrial function, at which point mitochondrial dysfunction becomes a major driver of hepatic inflammation. In addition to the ultrastructural abnormalities observed in MASLD, including paracrystalline inclusions, loss of cristae integrity, and impaired assembly of electron transport chain complexes, MASH is characterized by reduced respiratory capacity, diminished ATP synthesis, and increased oxidative damage affecting mitochondrial DNA, proteins, and lipids [14,15]. Importantly, several cellular stress-response and immune pathways implicated in MASH progression, including autophagy, mitophagy, the unfolded protein response, and antioxidant defense mechanisms, are themselves under circadian regulation [16,59,60,61].

Once circadian regulation is disrupted, the temporal coordination of these cellular programs is lost, leading to impaired mitochondrial quality control and the transition from tightly regulated adaptive responses to chronic and dysregulated activation. Mitophagy represents a critical component of this process. Indeed, PINK1-, Parkin-, BNIP3-, and FUNDC1-mediated mitophagy pathways are consistently downregulated in high-fat diet-induced murine models and, to a more limited and largely descriptive extent, in human MASLD/MASH liver tissue [61,62]. This results in the accumulation of damaged mitochondria, persistent release of mitochondrial damage-associated molecular patterns (mtDAMPs), and activation of pro-inflammatory and pro-fibrotic signaling pathways. Mitochondria-derived DAMPs, including mtDNA, mtRNA, ATP, and cardiolipin, activate Kupffer cells through multiple innate immune pathways, including TLR9, cGAS–STING signaling, and the NLRP3 inflammasome, ultimately promoting sterile hepatic inflammation [14,63,64].

Under physiological conditions, these responses are tightly regulated through circadian control of autophagy and antioxidant defense mechanisms. However, circadian desynchronization disrupts the rhythmicity of these protective pathways, resulting in sustained activation of innate immune signaling, recruitment of hepatic stellate cells, and progressive fibrogenesis [14,16,61]. Consistent with these observations, patients with MASLD/MASH exhibit elevated circulating levels of mtDNA, which correlate positively with the severity of hepatic inflammation and fibrosis [64,65].

It should be noted that the relationship between circadian disruption, mitochondrial dynamics, and MASLD/MASH pathogenesis outlined above is not strictly unidirectional, and several findings in the literature qualify this model. First, not all clock-gene perturbations reproduce a MASLD-like phenotype. Whole-body Bmal1 deletion is generally associated with increased steatosis susceptibility. By contrast, hepatocyte-specific Bmal1 deletion has been reported to reduce hepatic triglyceride content through enhanced lipolysis, reduced lipogenesis, and diminished lipid uptake, while concurrently impairing mitochondrial β-oxidation. This phenotype is therefore neither uniformly pro-steatotic nor unequivocally protective. Such discrepancies likely reflect differences in the tissue-specificity of the deletion, the feeding regimen, and compensatory input from extrahepatic clocks. Second, mitochondrial fission is not inherently pathological, and the consequences of restraining it appear to be disease-stage dependent. Early life inhibition of hepatocyte DRP1 has been reported to prevent high-fat diet-induced hepatic steatosis. By contrast, hepatocyte-specific Drp1 knockdown in mice with established NASH activates the mitochondrial integrated stress response and exacerbates inflammation, fibrosis, and hepatocyte death [35]. DRP1-mediated fission can therefore act as an adaptive response, and its inhibition does not consistently improve outcomes, contrary to a simple “fission-is-harmful” framework. Third, and conversely, a shift toward mitochondrial fusion is not uniformly protective. In the same model, Drp1 knockdown increases hepatocyte mitochondrial size while aggravating liver injury, indicating that unopposed fusion can itself become maladaptive [35]. Taken together, these findings indicate that mitochondrial dynamics are regulated within a physiological range in which both excessive fission and excessive fusion are potentially deleterious, and that the pathogenic relevance of a given morphological shift is context-, stage-, and stimulus-dependent rather than following a single directional rule.

A central mediator linking circadian regulation and mitochondrial function is the NAD^+^-sirtuin axis. Circadian oscillations in NAD^+^ levels are driven by rhythmic expression of NAMPT [66] and support the activity of both the nuclear deacetylase SIRT1 and the mitochondrial deacetylase SIRT3. Together, these enzymes regulate circadian clock amplitude and maintain mitochondrial metabolic function through the deacetylation of key enzymes involved in energy metabolism [67,68]. During both aging and MASLD progression, hepatic NAD^+^ levels progressively decline, resulting in attenuation of NAD^+^ rhythmicity and impaired SIRT3-dependent regulation of the mitochondrial acetylome [19,20]. Consequently, components of the OXPHOS system, TCA cycle enzymes, and proteins involved in long-chain fatty acid oxidation become hyperacetylated. These alterations are associated with reduced respiratory efficiency, accumulation of acyl-carnitines, and increased production of ROS, all of which have been documented in MASH liver tissue [14,15,19].

These events establish a self-perpetuating cycle in which circadian disruption impairs mitochondrial function, while mitochondrial dysfunction further weakens circadian regulation through reduced ATP- and NAD^+^-dependent signaling to AMPK and SIRT1 [6,7]. This reciprocal deterioration may explain why circadian and metabolic disorders frequently exacerbate one another over time and highlights the NAD^+^–sirtuin axis as a promising therapeutic target [19,20].

The clock–mitochondria axis also contributes to the broader metabolic complications associated with MASLD. Hepatic insulin resistance, a defining feature of MASLD and a major driver of disease progression, is exacerbated by circadian disruption. Liver-specific deletion of Bmal1 disrupts rhythmic gluconeogenesis and alters glucose homeostasis [30], whereas ClockΔ19 mutant mice develop hyperinsulinemia and glucose intolerance despite maintaining normal feeding behavior [52]. In addition, mitochondrial dysfunction directly contributes to insulin resistance through the accumulation of diacylglycerols and ceramides, which activate PKCε and promote inhibitory serine phosphorylation of the insulin receptor and IRS1, thereby impairing downstream insulin signaling [14,15].

At the advanced stage of disease progression, chronic circadian disruption has also been implicated in the development of hepatocellular carcinoma (HCC). ClockΔ19 mutant mice develop HCC spontaneously [53], while epidemiological studies have reported an increased incidence of HCC among shift workers, even after adjustment for dietary and lifestyle-related confounding factors [32,58]. Furthermore, experimental models of chronic jet lag have demonstrated that circadian disruption alone is sufficient to promote progression from NAFLD to HCC through mechanisms involving bile acid dysregulation and activation of the constitutive androstane receptor (CAR) [53]. Additional mechanisms linking circadian dysfunction to hepatocarcinogenesis include impaired clock-dependent DNA damage repair and sustained mitochondrial ROS production, both of which favor the accumulation of oncogenic mutations [14,53].

Interestingly, serum alanine aminotransferase (ALT), a widely used marker of liver injury, also exhibits a pronounced diurnal rhythm in patients with chronic liver disease, with significantly higher levels observed during the afternoon compared with the morning [22]. This observation further supports the concept that hepatic injury and disease progression are closely linked to the temporal organization of liver metabolism.

**Figure 2:**
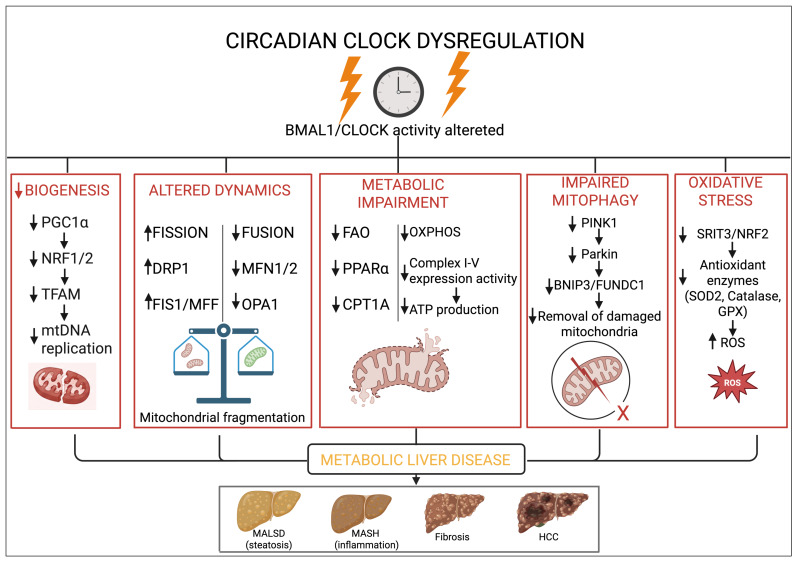
Schematic representation of circadian clock–mitochondria axis dysregulation as a driver of metabolic liver disease. Upward arrows (↑) indicate increased levels/activity, downward arrows (↓) indicate decreased levels/activity and downward arrows (↓) between sequential steps indicates progression to the downstream event. Created with Biorender.com.

Collectively, these findings demonstrate that the clock–mitochondria axis is not merely associated with MASLD progression but actively contributes to disease development across the entire spectrum, from simple steatosis and fibrosis to hepatocellular carcinoma (Figure 2). Consequently, this regulatory network represents a mechanistically relevant and potentially tractable target for therapeutic intervention.

## 6. Clock-Regulated Pathways as Targets for Chronotherapy in Metabolic Liver Disease

The identification of circadian regulation as a key determinant of hepatic mitochondrial function, together with evidence demonstrating that this temporal organization is disrupted in MASLD/MASH, provides a strong rationale for chronotherapeutic interventions.

Chronotherapy refers to the strategic timing of nutritional, behavioral, or pharmacological interventions to align with endogenous biological rhythms. While this approach has been successfully implemented in several areas of medicine, particularly cardiology and oncology, where it has demonstrated improvements in therapeutic efficacy, its application in hepatology remains largely unexplored [22].

A systematic analysis of more than 200,000 clinical trials registered on ClinicalTrials.gov revealed that only 0.16% incorporated a circadian-informed intervention, with none focusing on liver diseases [21]. This observation raises the possibility that some emerging therapies for MASLD may not achieve their full therapeutic potential because treatment administration is not synchronized with the circadian biology of their molecular targets. Although this hypothesis has not yet been directly evaluated in clinical trials of MASH, growing evidence supporting extensive circadian regulation of hepatic metabolism and mitochondrial function suggests that treatment timing may represent an important yet largely overlooked determinant of therapeutic response.

Accordingly, the following sections discuss several mitochondria-targeted or mitochondria-related interventions through the perspective of the clock–mitochondria axis. It should be noted, however, that although the circadian regulation of many of the molecular targets involved is well established, the clinical benefits of optimizing treatment timing remain to be rigorously evaluated in dedicated chronotherapeutic studies.

Table 1 summarizes the representative clock-regulated therapeutic compounds discussed in this section, together with their molecular targets, associated clock-controlled pathways, and current clinical development status.

Time-restricted eating (TRE), referred to as time-restricted feeding (TRF) in animal studies, represents one of the simplest and most accessible chronotherapeutic interventions. This approach involves restricting food intake to a defined daily window of approximately 8–12 h without reducing total caloric consumption. In murine models of diet-induced obesity, TRF has been shown to prevent hepatic steatosis, restore the rhythmic expression of clock-regulated metabolic genes, remodel hepatic transcriptomic and metabolomic profiles, and improve systemic insulin sensitivity, even when caloric intake is equivalent to that of ad libitum-fed controls [69,70].

At the mechanistic level, TRF restores the amplitude of hepatic NAD^+^ oscillations, reactivates SIRT1- and SIRT3-dependent signaling pathways, and re-establishes regulation of the mitochondrial acetylome. In addition, TRF promotes the recovery of rhythmic autophagy and mitophagy, facilitating the clearance of dysfunctional mitochondria and reducing inflammation driven by mitochondrial damage-associated molecular patterns (mtDAMPs) [59,70]. Collectively, these effects contribute to improved mitochondrial quality control and metabolic homeostasis.

Clinical studies are increasingly supporting the beneficial effects of TRE in patients with metabolic liver disease. The TREATY-FLD trial demonstrated that TRE significantly reduces hepatic fat content in individuals with NAFLD, with efficacy comparable to that achieved through caloric restriction [71]. More recently, a larger clinical study involving 337 patients with MASLD reported a 25.8% reduction in hepatic steatosis following 16 weeks of TRE, accompanied by significant decreases in body weight and waist circumference comparable to those observed with conventional caloric restriction strategies [72].

Importantly, the timing of food intake relative to the circadian cycle appears to influence therapeutic outcomes. Proof-of-concept studies in men with prediabetes demonstrated that early TRE, in which food consumption is concentrated during the morning and early afternoon, produces greater metabolic benefits than late TRE [73]. Translating these findings into a practical schedule, current evidence favors an early, active-phase-aligned feeding window over a late one. The protocols yielding the most consistent metabolic benefit in MASLD cohorts typically use 8–10 h windows initiated in the morning [71,72]. Based on this evidence, a reasonable chronopharmacological schedule would concentrate caloric intake between approximately 08:00 and 16:00–18:00. The largest meal would fall in the early-to-mid window (08:00–12:00), when insulin sensitivity and hepatic OXPHOS capacity are physiologically highest, followed by a lighter meal in the early afternoon (12:00–15:00). Caloric intake would cease by 16:00–18:00, followed by a 14–16 h overnight fast. This schedule is consistent with the early-TRF arm of the CHRONO-NAFLD trial, which combined a Mediterranean-type diet with a 14:10 early feeding window and achieved superior glycemic control compared with late feeding [74]. It should be emphasized that this schedule represents a rational extrapolation from the circadian biology and clinical trial designs reviewed above, rather than a validated clinical protocol. Adherence, individual chronotype, and occupational constraints are likely to significantly influence real-world feasibility and outcomes. These findings are consistent with the circadian regulation of insulin sensitivity and mitochondrial OXPHOS, both of which typically peak during the first half of the day. Therefore, TRE provides a compelling behavioral example of how restoring temporal alignment between nutrient intake and endogenous metabolic rhythms can improve mitochondrial function and metabolic health. Nevertheless, long-term adherence, as well as the optimal timing and duration of the feeding window across different populations, remains to be determined.

Hepatic NAD^+^ levels are reduced in MASLD and decline further with aging, and this depletion contributes to impaired SIRT3-dependent mitochondrial quality control.

Pharmacological restoration of NAD^+^ homeostasis has emerged as a promising therapeutic strategy for MASLD, supported by substantial preclinical evidence. Supplementation with NAD^+^ precursors, particularly nicotinamide riboside (NR) and nicotinamide mononucleotide (NMN), has been shown to restore hepatic NAD^+^ levels, reactivate SIRT3-dependent pathways, reduce protein hyperacetylation, and improve mitochondrial OXPHOS efficiency and fatty acid β-oxidation in experimental models of MASLD [19,20]. Early clinical studies in obese individuals demonstrated that NR is well-tolerated and effectively increases circulating NAD^+^ concentrations [75].

More disease-specific evidence has subsequently emerged from clinical investigations in patients with NAFLD. A double-blind, placebo-controlled trial evaluating a combination of NR and pterostilbene (NRPT) reported reductions in markers of hepatic inflammation [76]. Similarly, a phase II study investigating a combined metabolic activator formulation containing NR demonstrated an approximately 10% reduction in hepatic fat content in patients with NAFLD [77]. Although these findings are encouraging, the available clinical evidence remains preliminary, and larger randomized studies incorporating histological endpoints are required before NAD^+^ supplementation can be considered an established therapeutic approach.

An additional consideration is the circadian regulation of NAD^+^ metabolism itself. Endogenous NAD^+^ levels exhibit robust daily oscillations and typically reach peak concentrations during the active phase under the control of rhythmically expressed NAMPT [66]. Consequently, the timing of NAD^+^ precursor administration may influence therapeutic efficacy by altering interactions with endogenous NAD^+^ rhythms. For example, morning administration may reinforce the physiologically active-phase NAD^+^ peak, whereas evening supplementation could potentially interfere with the declining phase of the rhythm. This rationale can be extended into a practical schedule, although it remains untested in dedicated chronopharmacological trials. NAD^+^ precursor supplementation (NR or NMN) would be expected to align best with the endogenous NAMPT-driven NAD^+^ peak when administered in the morning, approximately between 07:00 and 09:00 [78]. Ideally, it should be taken with or shortly after breakfast to coincide with the onset of the active phase and peak hepatic metabolic demand. A second, smaller dose in the early afternoon (13:00–14:00) could theoretically sustain NAD^+^ availability through the peak activity window without encroaching on the physiological evening decline. Administration in the late evening is not recommended on theoretical grounds, as it would misalign supplementation with the natural NAD^+^ trough and could blunt the amplitude of the endogenous rhythm rather than reinforce it. While this concept remains hypothetical, it represents an important area for future chronopharmacological investigation.

More broadly, many pharmacological approaches currently under development for MASLD target metabolic pathways that are themselves under circadian regulation. Among these, peroxisome proliferator-activated receptors (PPARs) represent key regulators of hepatic lipid metabolism. Specifically, PPARα and PPARδ serve as major transcriptional drivers of fatty acid β-oxidation through the induction of genes encoding carnitine palmitoyltransferase 1 (CPT1), acyl-CoA dehydrogenases, and other enzymes involved in mitochondrial fatty acid catabolism. In addition, PPAR activation promotes mitochondrial biogenesis through PGC-1α signaling [22,29].

Importantly, all three PPAR isoforms exhibit circadian patterns of expression in the liver and are regulated by core clock components, including CLOCK, BMAL1, and REV-ERBα [22,79,80]. Furthermore, genetic deletion of PPARα disrupts the rhythmic expression of clock genes and impairs circadian adaptation to feeding schedules [22,81]. Evidence from preclinical studies also indicates that the biological activity of PPAR agonists is time-dependent. For instance, nocturnal administration of bezafibrate induces hepatic FGF21 expression, whereas administration during the morning fails to produce a comparable response [82].

These observations suggest that pharmacological agents currently under investigation for MASH, including the dual PPARα/δ agonist elafibranor and the pan-PPAR agonist lanifibranor, may exhibit enhanced efficacy when administered during periods of increased hepatic fatty acid flux and β-oxidation demand, corresponding to the active phase of the circadian cycle [22,83]. Whether such chronopharmacological optimization translates into clinically meaningful benefits remains to be determined.

Another promising therapeutic strategy involves inhibition of acetyl-CoA carboxylase (ACC), a key enzyme responsible for producing malonyl-CoA, a potent endogenous inhibitor of CPT1. By reducing malonyl-CoA availability, ACC inhibition enhances mitochondrial fatty acid transport and β-oxidation. Firsocostat, a dual ACC1/ACC2 inhibitor, has demonstrated efficacy in reducing hepatic fat accumulation and improving fibrosis-associated biomarkers in clinical trials involving patients with NASH [84].

Acetyl-CoA carboxylase (ACC) is also subject to circadian regulation, with hepatic ACC expression exhibiting a daily rhythmicity that is partially controlled by the clock-regulated histone deacetylase HDAC3 [85]. Furthermore, numerous plasma lipid species display circadian oscillations in humans, reflecting the temporal organization of lipid metabolism [86]. Interestingly, one of the major on-target adverse effects associated with ACC inhibition—elevated plasma triglyceride levels resulting from reduced PPARα activity and enhanced SREBP1c-mediated lipogenesis—also involves pathways that are under circadian control [87]. Consequently, optimizing the timing of ACC inhibitor administration may represent a potential strategy to minimize this adverse effect. However, this possibility remains speculative, as no clinical studies have yet evaluated the impact of dosing time on the efficacy or safety of ACC inhibitors.

Among the therapeutic candidates currently under investigation for MASH, MSDC-0602K possesses one of the most direct mitochondria-targeted mechanisms of action. This compound modulates the mitochondrial pyruvate carrier (MPC), thereby reducing pyruvate transport into the mitochondrial matrix and redirecting glucose-derived carbon away from mitochondrial oxidation toward alternative metabolic pathways. Improved insulin sensitivity represents the primary therapeutic outcome associated with this mechanism [22,88]. Results from a phase IIb clinical trial demonstrated improvements in insulin resistance, hepatic glucose production, and markers of liver injury, without the fluid retention and weight gain commonly associated with full PPARγ agonists [88].

Given that the MPC occupies a critical position at the interface between glycolysis and the TCA cycle, and considering that several key enzymes involved in pyruvate metabolism exhibit PER1/PER2-dependent circadian oscillations [89], the efficacy of MSDC-0602K may depend on the temporal state of hepatic metabolic pathways. Consequently, chronopharmacological optimization represents a potentially valuable approach for enhancing therapeutic efficacy; however, this concept has not yet been investigated experimentally or clinically.

A major milestone in the treatment of MASH was achieved in March 2024 with the FDA approval of resmetirom (Rezdiffra), the first therapy specifically approved for MASH based on the results of the MAESTRO-NASH phase III clinical trial [90,91]. Resmetirom is a liver-targeted, oral thyroid hormone receptor-β (THR-β) selective agonist that promotes hepatic fatty acid oxidation and reduces lipotoxicity. Activation of THR-β signaling stimulates mitochondrial biogenesis, enhances β-oxidation, and promotes mitophagy, thereby improving mitochondrial function and metabolic homeostasis.

Importantly, the hypothalamic–pituitary–thyroid (HPT) axis is itself under circadian regulation, with thyroid-stimulating hormone (TSH) and thyroid hormone concentrations displaying pronounced diurnal fluctuations [22]. Clinical studies have shown that evening administration of thyroxine in hypothyroid patients results in higher circulating thyroid hormone levels and lower TSH concentrations compared with morning dosing [22]. 

Therefore, part of the pharmacodynamic variability observed among patients receiving resmetirom may reflect differences in the timing of drug administration. Whether chronopharmacological optimization of THR-β agonist therapy could enhance efficacy or reduce interindividual variability remains an important unanswered question.

Another promising target at the intersection of circadian and mitochondrial biology is fibroblast growth factor 21 (FGF21). FGF21 functions as a hepatokine that is induced by mitochondrial stress, fasting, and oxidative stress, and subsequently promotes fatty acid oxidation, mitochondrial biogenesis through PGC-1α, and systemic metabolic adaptation in peripheral tissues and the central nervous system [22,29,92]. In humans, circulating FGF21 concentrations exhibit a pronounced circadian rhythm characterized by a peak during the early morning hours followed by a marked decline throughout the day. Notably, this rhythmic pattern is attenuated in obesity and metabolic disorders [22,93].

Beyond its peripheral metabolic functions, FGF21 can cross the blood–brain barrier and modulate circadian behavior through actions on β-Klotho-expressing neurons within the SCN [94]. This observation highlights a bidirectional communication pathway through which mitochondrial status in peripheral tissues can influence central circadian regulation. The FGF21 analog pegbelfermin has demonstrated efficacy in reducing hepatic fat accumulation in patients with MASH when administered as a weekly subcutaneous injection. However, this treatment results in sustained elevations of circulating FGF21 that differ substantially from its physiological rhythmic pattern [22,95].

It has been proposed that chronic elevation of FGF21 may promote receptor desensitization and potentially impair adaptive mitochondrial responses [22,93]. Consequently, therapeutic strategies that restore physiological FGF21 rhythmicity rather than continuously elevating hormone levels may provide superior long-term metabolic benefits. Comparative studies directly evaluating these approaches would therefore be of considerable interest.

Evidence supporting chronotherapy also comes from several other medical disciplines. For example, short-acting statins are routinely administered in the evening to coincide with the nocturnal peak in HMG-CoA reductase activity, thereby maximizing their inhibitory effects on cholesterol biosynthesis while also influencing the production of mitochondrial ubiquinone [96]. Similarly, evening administration of certain cytotoxic chemotherapeutic agents has been associated with improved outcomes in pediatric acute lymphoblastic leukemia, partly due to circadian variations in enzymes involved in mitochondrial DNA replication and DNA repair pathways [97,98]. Furthermore, formal guidelines for chronopharmacology-based clinical trials have already been established in the field of hypertension management [99].

Taken together, these examples demonstrate the feasibility and potential clinical value of aligning therapeutic interventions with endogenous biological rhythms. Given the extensive interplay between circadian regulation, mitochondrial function, and hepatic metabolism, mitochondria-centered chronotherapy represents a promising strategy for the management of MASLD/MASH and warrants further evaluation in dedicated clinical studies.

### Chronobiotics: Direct Pharmacological Modulation of the Core Molecular Clock

A pharmacologically distinct strategy, conceptually different from the interventions discussed above, involves direct modulation of the core molecular clock machinery itself rather than of downstream clock-regulated metabolic pathways. Compounds of this class, termed chronobiotics, act directly on core clock components such as REV-ERBα/β, RORα/γ, or cryptochrome (CRY), thereby altering the amplitude, phase, or period of the circadian oscillator, rather than modulating a pathway that the clock merely regulates.

REV-ERBα/β agonists, including SR9009 and SR9011, have demonstrated protective effects against hepatic steatosis, insulin resistance, inflammation, and fibrosis in diet-induced murine models of NASH, with efficacy that varies according to the time of administration [100]. Notably, recent work has extended this pharmacology beyond metabolic disease, showing that SR9009 can potentiate the antitumor efficacy of sorafenib in hepatocellular carcinoma by disrupting tumor mitochondrial metabolism [101]. The RORα/γ agonist nobiletin similarly enhances circadian amplitude and protects against diet-induced metabolic syndrome in a clock gene-dependent manner, in part through activation of hepatic RORα/γ target genes [102]. The cryptochrome stabilizer KL001 prevents ubiquitin-dependent degradation of CRY, thereby lengthening the circadian period, and inhibits glucagon-induced gluconeogenesis in primary hepatocytes [103].

**Table 1:** Representative clock-regulated therapeutic compounds evaluated in metabolic liver disease, summarizing their molecular targets, associated clock-controlled pathways, and current development status. Upward arrows (↑) indicate increased levels/activity, downward arrows (↓) indicate decreased levels/activity, right arrows → indicates activation of the downstream pathway/process.

Compound	Target/Mechanism	Clock-Regulated Pathways	Clinical Status
Time-restricted eating (TRE)	Nutritional chronotherapy → clock re-entrainment, NAD^+^ rhythm restoration, mitophagy	Feeding-sensitive hepatic clock; SIRT1/SIRT3; circadian autophagy	Clinical evidence: TREATY-FLD [71], Sutton, E.F et al. 2018 [73]
Nicotinamide riboside (NR)	NAD^+^ precursor → SIRT1/SIRT3 activation	NAMPT-NAD^+^ oscillations; circadian acetylome	Phase II (NAFLD) [75,76]
Nicotinamide mononucleotide (NMN)	NAD^+^ precursor → SIRT3 reactivation, mitochondrial QC	NAMPT-NAD^+^ oscillations	Phase I/II [78]
Elafibranor (dual PPARα/δ agonist)	PPARα/δ → β-oxidation, mitochondrial biogenesis	PPARα circadian expression; BMAL1/REV-ERBα	Phase III (NASH)-development discontinued [83]
Lanifibranor (pan-PPAR agonist)	PPARα/δ/γ → lipid metabolism, inflammation	Pan-PPAR circadian expression; clock–lipid axis	Phase III (MASH)-ongoing [83]
Firsocostat (GS-0976)	ACC1/ACC2 inhibitor → ↓ malonyl-CoA → ↑β-oxidation	HDAC3-controlled ACC circadian rhythmicity [84]	Phase II (NASH) [84]
MSDC-0602K	Mitochondrial pyruvate carrier (MPC) modulator → insulin sensitization	PER1/PER2-dependent pyruvate enzyme oscillations [88]	Phase IIb (NASH)-completed [88]
Resmetirom (Rezdiffra)	THR-β agonist → hepatic FAO, mitophagy, mitochondrial biogenesis	HPT axis circadian regulation; diurnal TSH/T3 fluctuations [22]	FDA-approved (MASH with fibrosis, March 2024) [90,91]
Pegbelfermin (FGF21 analog)	FGF21 → PGC-1α, mitochondrial biogenesis, FAO	Circadian FGF21 rhythm (morning peak); SCN modulation [92,93]	Phase 2a (MASH)-completed [95]
Semaglutide (GLP-1RA)	GLP-1R agonist → hepatic fat, inflammation	Clock-regulated GLP-1R expression; circadian insulin sensitivity	Phase III (ESSENCE)-positive results 2025 [100]
Nobiletin	Direct clock modulator → ROR agonist (RORα/RORγ)	downstream AMPK–SIRT1 signaling	Preclinical (chronobiotic) [102]
SR9009/SR9011 (REV-ERBα/β agonists)	Direct clock modulator → ↓ lipogenesis, ↑ FAO, mitochondrial biogenesis	Core TTFL (REV-ERBα/β); mitochondrial biogenesis via PGC-1α [101,102]	Preclinical (chronobiotic) [103]
KL001	Direct clock modulator → CRY1/CRY2 stabilizer	CRY-mediated repression of CLOCK/BMAL1; suppresses hepatic gluconeogenesis	Preclinical (chronobiotic) [104]

Collectively, these compounds illustrate that pharmacologically reinforcing or resetting the core oscillator, rather than acting on its downstream outputs, can favorably influence hepatic and mitochondrial metabolism. However, chronobiotics remain at an entirely preclinical stage of development. SR9009 has been associated with dose-dependent hepatotoxicity in animal studies, and its interpretation as a selective REV-ERB agonist has been further complicated by evidence that it exerts substantial REV-ERB-independent effects on cell proliferation and mitochondrial metabolism, including in REV-ERBα/β double-knockout cells [74]. This off-target activity raises important caveats regarding the mechanistic attribution of SR9009 effects reported in hepatic and oncological models. More broadly, none of these compounds have entered human clinical trials, and their safety, target specificity, and pharmacokinetic profile in the human liver remain to be established. As such, chronobiotics currently represent a proof-of-concept pharmacological strategy rather than a translatable therapeutic option, though they underscore the broader principle that the circadian clock itself, and not only its downstream targets, constitutes a viable point of pharmacological intervention.

## 7. Outstanding Questions and Future Perspectives

The clock–mitochondria axis in the liver remains a relatively young field of investigation, and several clinically important questions remain unresolved. Although substantial progress has been made in understanding the molecular interplay between circadian regulation and mitochondrial function, significant gaps in knowledge continue to limit translational applications. One of the most important challenges concerns the current state of direct evidence demonstrating circadian regulation of mitochondrial dynamics and mitophagy in human hepatic cells. Autonomous circadian transcriptional and functional rhythms have recently been documented directly in primary human hepatocytes, with demonstrated impact on drug metabolism and inflammatory signaling [101]. More directly relevant to the mitochondrial dimension, BMAL1-dependent circadian oscillations in mitochondrial network dynamics and autophagic activity have recently been reported in a human hepatocellular carcinoma cell line (HepG2) [102]. However, direct evidence remains lacking for non-transformed primary human hepatocytes and for intact human liver tissue in vivo. Consequently, it remains unclear whether processes such as DRP1 phosphorylation, mitochondrial network remodeling, and selective mitophagy are disrupted in MASLD/MASH. Time-of-day-stratified liver biopsy studies, ideally paired with single-cell or organelle-resolved and mitophagy-selective readouts, could provide critical evidence to establish the translational relevance of these findings.

Another important area of investigation concerns the extent to which clock–mitochondria coupling is conserved between rodents and humans. While rodents are nocturnal and humans are diurnal, resulting in opposite phases of many circadian processes, it remains uncertain whether the underlying molecular mechanisms are fully conserved or whether species-specific regulatory features exist. Comparative chronobiological studies, supported by ex vivo analyses of human liver tissue, will be necessary to clarify the similarities and differences between these systems and to improve the interpretation of findings derived from animal models.

At the same time, the development of reliable biomarkers of circadian function represents a major prerequisite for the implementation of chronotherapy in clinical practice. Without accurate methods for assessing circadian disruption in individual patients, the evaluation of chronotherapeutic interventions in MASH will remain challenging. Potential biomarkers include dim-light melatonin onset (DLMO), diurnal cortisol profiles, time-resolved serum metabolomic and lipidomic signatures, and rhythmic expression of clock genes in peripheral blood cells. However, whether these surrogate markers accurately reflect hepatic circadian activity and whether their normalization predicts therapeutic responses remains an important unanswered question.

The rapid expansion of the therapeutic landscape for MASH further highlights the need to integrate chronobiological considerations into clinical trial design. The recent approval of resmetirom, the positive results obtained with semaglutide in the phase III ESSENCE trial, the continued development of lanifibranor, and the increasing likelihood of combination therapies all raise important questions regarding the optimal timing of treatment administration. Because many of these agents target pathways that are under circadian regulation, their efficacy and safety profiles may vary according to dosing time. Clinical studies that incorporate treatment timing as a predefined variable, ideally in conjunction with chronotype-based stratification, will be essential for determining whether current dosing strategies can be further optimized.

An additional area that remains largely unexplored is the interaction between circadian regulation and the spatial organization of hepatic metabolism. Hepatocytes exhibit considerable metabolic heterogeneity across the hepatic lobule, yet little is known about how mitochondrial function and circadian programs differ among distinct hepatocyte populations.

Emerging single-cell and single-nucleus RNA sequencing approaches have already resolved substantial zonation-dependent heterogeneity in hepatocyte metabolic potential and gene expression [48,49]. These approaches could analogously identify zonation-specific circadian transcriptional programs and their coupling to mitochondrial gene expression at single-cell resolution. Complementary spatial transcriptomic platforms have recently mapped cell-type and zonal heterogeneity in healthy and fibrotic human liver at single-cell resolution [105]. Such platforms could be extended to map mitochondrial morphology and quality-control markers directly onto the periportal–pericentral axis, testing whether circadian mitochondrial remodeling is uniform across the lobule or restricted to specific zones. At the organelle level, genetically encoded mitophagy reporters such as mito-QC and mt-Keima enable quantitative, tissue-resolved assessment of mitophagic flux in vivo [106]. Combined with time-of-day-stratified sampling, these reporters could provide direct readouts of mitophagy rhythmicity, rather than the static, correlative markers used in most human studies to date. Finally, patient-derived liver organoids and precision-cut liver slices, sampled longitudinally under controlled light–dark or feeding schedules, offer an ethically tractable alternative to serial human biopsy. This approach could test whether the circadian–mitochondrial mechanisms established in rodents are conserved in human hepatic tissue. Integrating these technologies with the time-of-day-stratified biopsy studies discussed above represents a concrete, technically feasible path toward closing the translational gap identified throughout this review.

The integration of temporal information into these emerging omics and imaging approaches may help determine whether MASLD progression is associated with disruption of both temporal and spatial metabolic organization.

Although these challenges represent only a subset of the unanswered questions in the field, they collectively define a research framework that integrates chronobiology, mitochondrial biology, hepatology, and pharmacology. Addressing these knowledge gaps has the potential to substantially advance our understanding of liver disease pathogenesis and facilitate the development of more effective, mechanism-based therapeutic strategies in the coming years.

## 8. Conclusions

The hepatic circadian clock and the mitochondrial network function as a highly integrated regulatory system rather than as independent cellular components. Within this framework, the circadian clock orchestrates daily fluctuations in mitochondrial morphology, bioenergetic activity, and quality control mechanisms through processes involving rhythmic DRP1 phosphorylation and regulation of the NAD^+^–sirtuin axis. In turn, mitochondrial metabolism provides feedback signals that influence both the amplitude and periodicity of the molecular clock. This bidirectional interaction enables hepatocytes to anticipate recurring feeding–fasting cycles and to synchronize mitochondrial energy production with predictable metabolic demands.

Disruption of this coordinated network has profound consequences for liver physiology and contributes to disease progression across the entire spectrum of metabolic liver disorders, ranging from simple steatosis to MASLD/MASH, fibrosis, and ultimately hepatocellular carcinoma (HCC). Increasing evidence indicates that dysfunction of the clock–mitochondria axis is not merely associated with these conditions but actively participates in their pathogenesis through alterations in mitochondrial dynamics, oxidative metabolism, mitophagy, and inflammatory signaling pathways.

Importantly, the therapeutic implications of these findings are becoming increasingly apparent. Several interventions currently used or under development for MASH, including time-restricted eating, NAD^+^ repletion strategies, PPAR agonists, ACC inhibitors, mitochondrial pyruvate carrier modulators, the THR-β agonist resmetirom, and FGF21-based therapies, target pathways that are subject to significant circadian regulation. Consequently, these approaches offer an opportunity to exploit the temporal organization of hepatic metabolism and mitochondrial function for therapeutic benefit.

Given the pronounced rhythmicity of many pathways targeted by current MASH therapies, the limited consideration of treatment timing may represent an overlooked factor contributing to the modest and sometimes variable efficacy observed in clinical trials of otherwise promising compounds. Future clinical studies should therefore consider incorporating dosing schedules aligned with endogenous mitochondrial and metabolic rhythms, treating treatment timing as a predefined experimental variable rather than a secondary consideration.

More broadly, the integration of chronobiological principles into hepatology, from basic mechanistic research to clinical practice and trial design, has the potential to fundamentally advance our understanding of metabolic liver disease and improve therapeutic outcomes. As knowledge of the clock–mitochondria axis continues to expand, mitochondria-centered chronotherapy may emerge as an important component of precision medicine strategies for the prevention and treatment of MASLD/MASH.

## Data Availability

No new data were created or analyzed in this study. Data sharing is not applicable to this article.

## Abbreviations

SCN, suprachiasmatic nucleus; TTFL, transcription–translation feedback loop; CLOCK, circadian locomotor output cycles kaput; NPAS2, neuronal PAS domain protein 2; BMAL1, brain and muscle ARNT-like 1; PER, Period; CRY, Cryptochrome; FBXW11/FBXL3/FBXL21, F-box and WD/leucine-rich repeat protein; REV-ERBα/β (NR1D1/NR1D2), nuclear receptor subfamily 1 group D member 1/2; ROR, RAR-related orphan receptor; RRE, ROR–REV-ERB response element; SIRT1/SIRT3, sirtuin 1/3; NAMPT, nicotinamide phosphoribosyltransferase; NAD^+^, nicotinamide adenine dinucleotide; AMPK, AMP-activated protein kinase; PGC-1α, peroxisome proliferator-activated receptor gamma coactivator 1-alpha; GLUT2, glucose transporter 2; NAFLD, non-alcoholic fatty liver disease; MASH, metabolic dysfunction-associated steatohepatitis; T2D, type 2 diabetes; MFN1/2, mitofusin 1/2; OPA1, optic atrophy 1; OXPHOS, oxidative phosphorylation; DRP1, dynamin-related protein 1; PKA, protein kinase A; CT, circadian time; GSH/GSSG, reduced/oxidized glutathione; ATP, adenosine triphosphate; EV, extracellular vesicle; mitoEV, mitochondrial extracellular vesicle; MVB, multivesicular body; MDV, mitochondria-derived vesicle; PINK1, PTEN-induced kinase 1; TOM20, translocase of outer mitochondrial membrane 20; LC3, microtubule-associated protein light chain 3; MID49/MID51, mitochondrial dynamics protein of 49/51 kDa; MFF, mitochondrial fission factor; STX17, syntaxin-17; Vps35, vacuolar protein sorting 35; MAPL, mitochondrial-anchored protein ligase; SNX9, sorting nexin 9; CD38, cluster of differentiation 38; cADPR, cyclic ADP ribose; VDAC1, voltage-dependent anion channel 1; HSP60/HSP70/mtHSP70, heat shock protein 60/70/mitochondrial 70; SOD2, superoxide dismutase 2; TCA, tricarboxylic acid; mtDNA, mitochondrial DNA; mtRNA, mitochondrial RNA; miRNA, microRNA; ARRDC1, arrestin domain-containing protein 1; mGluR3, metabotropic glutamate receptor 3; MISEV, Minimal Information for Studies of Extracellular Vesicles; TMRM, tetramethylrhodamine methyl ester.
